# Translation of a Mediterranean-Style Diet into the Australian Dietary Guidelines: A Nutritional, Ecological and Environmental Perspective

**DOI:** 10.3390/nu11102507

**Published:** 2019-10-18

**Authors:** Evangeline Mantzioris, Anthony Villani

**Affiliations:** 1 School of Pharmacy and Medical Sciences, Alliance for Research in Exercise, Nutrition and Activity (ARENA), University of South Australia, Adelaide 5005, Australia; evangeline.mantzioris@unisa.edu.au; 2 School of Health and Sport Sciences, University of the Sunshine Coast, Sippy Downs, QLD 4072, Australia

**Keywords:** Mediterranean diet, dietary guidelines, Australia, environmental sustainability, Mediterranean diet adherence

## Abstract

A Mediterranean diet (MedDiet) has been widely investigated and promoted as one of the ‘healthiest’ dietary patterns with respect to reductions in chronic disease risk and longevity. Moreover, it also emphasizes a plant-based dietary pattern consistent with an environmentally sustainable healthy reference diet conveyed by the EAT-Lancet Commission report. Nevertheless, the MedDiet does not exclude, but rather moderates consumption of animal-based foods, and therefore has emerged as a dietary pattern that could address both health and environmental concerns. However, whether non-Mediterranean countries such as Australia can adhere to such dietary principles is less clear. In this narrative review, we present evidence from eight randomized control trials conducted in Australia which demonstrates impressive and sustained adherence to a MedDiet intervention. However, we also report heterogeneity in the dietary protocols and prescriptive interpretation of a MedDiet across all studies presented in this review, making interpretations of the efficacy and adherence challenging. Based on the observable health benefits, translating key dietary elements of a Mediterranean-style diet within the Australian population remains attractive. However, adapting or modernizing traditional dietary patterns to satisfy the population’s nutritional requirements and/or acceptability warrants further exploration.

## 1. Introduction

The Australian Dietary Guidelines (ADGs) provides information on the types and amounts of food to eat from a range of core food groups and emphasizes a dietary pattern to promote health and wellbeing and reduce overall risk of non-communicable diseases (NCDs). However, a large portion of NCDs in Australia are attributable to lifestyle-related risk factors, including poor dietary behaviors [1]. Data taken from the 2015 Global Burden of Disease study revealed that one-fifth of NCD deaths were attributable to dietary risk factors [2]. Specifically, low consumption of fruits, vegetables, nuts and seeds, and wholegrains, coupled with a high sodium intake were the major dietary contributors to both NCD deaths and disability-adjusted life years (DALYs) [2]. Moreover, rates of overweight and obesity in Australia have been steadily climbing [3]. In 2017–2018, 67% of adults were classified as overweight or obese [4], with predictive modelling suggesting significant increases in the prevalence of severe obesity (BMI  >  35) by 2025 [5].

The paradigm of assessing dietary patterns as opposed to individual nutrients or single foods as a determinant of overall health and disease risk has been recognized for some time [6]. Food-based dietary guidelines attempt to translate a vast evidence base regarding the relationship between foods, dietary patterns, and health related outcomes into specific, culturally acceptable and actionable dietary recommendations [7]. Given the recent dietary trends supporting that the consumption of nutrient-dense food groups in Australia is markedly lower than current dietary recommendations [8,9], it would appear that the ADGs are ‘loosely’ followed and their translational potential may need to be revisited.

Over the past several decades, the Mediterranean Diet (MedDiet) has been widely investigated and promoted as one of the ‘healthiest’ dietary patterns with respect to reductions in chronic disease risk and longevity [10,11]. However, the majority of evidence from clinical trials assessing the efficacy of the MedDiet on health-related outcomes has been conducted in Mediterranean populations, where the dietary pattern and other non-dietary customs are familiar [12,13].

Although the transferability of a Mediterranean style diet in non-Mediterranean regions is appealing [14] and has been of interest to researchers for some time [15,16], embedding principles of a MedDiet in non-Mediterranean populations with different cultural food habits and cultural customs is potentially challenging. In a sample of middle-aged adults from the United Kingdom, Middleton et al. [15] reported that participants identified a number of perceived barriers when attempting to adapt principles of the MedDiet, including purchasing, organizing and preparing food due to time pressures for those in full-time employment. Moreover, the authors also cited cultural and lifestyle barriers encountered by non-Mediterranean populations as likely obstacles toward achieving adherence to a MedDiet [15]. Nevertheless, given the multiethnic landscape of Australia, coupled with the high prevalence of NCDs and the scientific evidence base of the MedDiet, assessing whether the Australian population can adhere to principles of a MedDiet is warranted. However, to the best of our knowledge, systematic evaluation describing adherence patterns to a MedDiet across clinical trials conducted in Australia is scant.

Therefore, in this narrative review we will aim to explore the efficacy and adherence to a MedDiet in clinical trials conducted in Australia. Peer-reviewed literature included in this narrative review was identified through searches of publications listed in electronic databases including MEDLINE, EMBASE, OVID, and PubMED. All studies included in this review were randomized control trials (RCTs), conducted in Australia, investigating the efficacy of a MedDiet against any disease outcome, in men and/or women aged ≥18 years.

## 2. The Traditional Mediterranean Diet: A Win-Win Dietary Pattern?

The traditional MedDiet is the dietary pattern prevailing among the people of the olive growing regions of the Mediterranean basin before the mid-1960s. The proposed health benefits of the MedDiet, and its identity, is partly attributed to the consumption of traditional foods, which are critical components of this dietary pattern [17]. We recently reviewed the literature and identified a number of dietary constituents and non-nutritional behaviours which define a traditional MedDiet pattern [18].

To attain a greater understanding of the mechanisms associated with the proposed health benefits of the MedDiet, adherence to the dietary pattern must be quantified. Over the past several years, several MedDiet index tools have been published within the literature for assessing overall adherence to a MedDiet. Among the most prominent methods used to assess dietary patterns are *a priori* numerical indices, which measure adherence to a dietary pattern that has been pre-defined on the basis of previous scientific evidence [19]. Two of the most pertinent scoring systems that have been used to operationalize the MedDiet include: (1) The Mediterranean Diet Score (MDS) developed by Trichopoulou et al. [20] and (2) the 14-item Mediterranean Diet Adherence Screener (MEDAS) [21] used in the largest clinical trial conducted investigating the efficacy of the MedDiet on cardiovascular disease (CVD) risk, the PREDIMED (PREvencion con DIeta MEDiterranea) study [22]. Nevertheless, regardless of the operationalization of adherence to a MedDiet, interpretation of a MedDiet is often characterized by a high intake of vegetables, fruits, nuts, legumes, unprocessed cereals, and daily use of extra-virgin olive oil (EVOO) incorporated to all meals, particularly composite dishes of vegetables and legumes (3–4 tablespoons per day); a moderate consumption of fish, shellfish and fermented dairy products (cheese and yogurt); a low consumption of meat and meat products, processed cereals, sweets, vegetable oils and butter; and the consumption of wine, typically during meals [23]. Moreover, the dietary pattern offers a unique nutritional profile rich in monounsaturated and omega-3 (*n*-3) fatty acids, vitamins, minerals, dietary fiber, and non-nutritive compounds including polyphenols, carotenoids and flavonoids [24].

**Figure 1:**
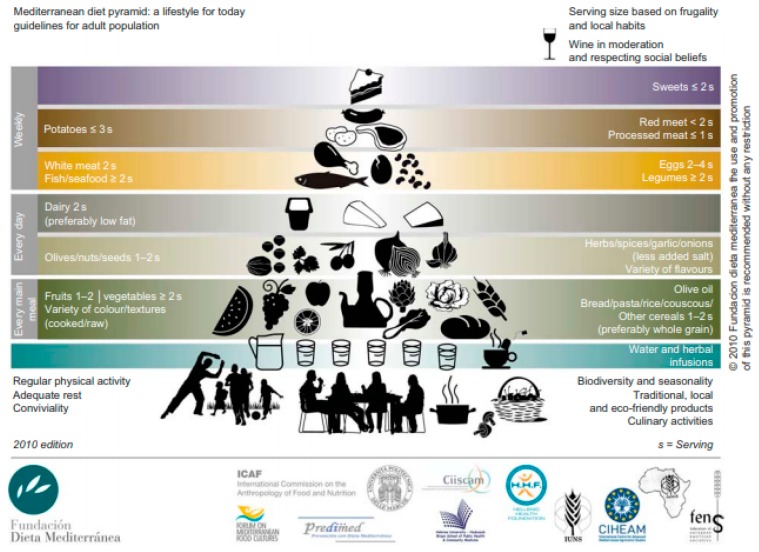
Mediterranean diet pyramid: A lifestyle for today [24].

Consumption of a wide variety of fresh and local products coupled with traditional culinary practices, frugality and conviviality, represents the cornerstone of a traditional MedDiet pattern (Figure 1) [17,24]. From an environmental perspective, production of traditional foods consistent with a traditional MedDiet pattern typically occurs using sustainable approaches, contributing to rural development and the preservation of biodiversity [25,26]. Moreover, given that frugality is an overarching principle of the MedDiet, emphasis is placed on food preparation methods, harvesting, conservation, picking, fishing, animal husbandry, moderation of portion size, sharing and avoiding food waste [26]. However, due the globalization of food production and adoption of western-type dietary behaviors, recent evidence suggests that adherence to a MedDiet has declined around the world [27,28,29]. This has also been partly due to the intensification and industrialization of agricultural systems taking precedence over sustainable food systems and human ecology [27,28,29]. A landmark publication acknowledging the need for healthy and sustainable food systems, the ‘Food in the Anthropocene: The EAT-Lancet Commission on healthy diets from sustainable food systems’ report [30] focuses mainly on environmental sustainability of food production and the health consequences of final consumption.

Referred to as a ‘win-win’ dietary pattern for population health and environmental sustainability, key findings from the EAT-Lancet Commission report emphasize the need to transform to a universal ‘healthy reference diet’ or ‘flexitarian’ diet by 2050 [30]. However, achieving a global transformation to a healthy reference diet will require substantial dietary shifts, including significant reductions in the global consumption of red meat and sugar, and significant increases in consumption of plant-based foods, such as nuts, fruits, vegetables, and legumes [30,31]. For these reasons, interest in the MedDiet as a plant-centered dietary pattern that does not exclude, but rather moderates consumption of animal-based foods, has emerged as a ‘win-win’ dietary pattern that could address both health and environmental concerns [27].

## 3. Efficacy on Health-Related Primary Outcomes Using the Mediterranean Diet as a Dietary Intervention in Clinical Trials conducted in Australia: What Is the Evidence?

Although the MedDiet is one of the most widely reported dietary patterns, few Australian studies have explored the proposed health benefits and levels of adherence using robust clinical trials. In this narrative review, we report on a total of eight RCTs conducted in Australia, published between 2011 and 2019 with sample sizes ranging from 27 to 166 participants against primary outcomes related to cardiometabolic risk factors, glycaemic control, cognition, hepatic steatosis and depressive symptomology. Study participants included patients with well-controlled type-2 diabetes mellitus (T2DM), pre-existing CVD, middle-aged to older adults with CVD risk factors, patients with diagnosed non-alcoholic fatty liver disease (NAFLD) and depression (Table 1).

One of the earliest studies reported on Australian participants was conducted by Itsiopoulos et al. [32] who showed statistical and clinically significant improvements in glycemic control (HbA1c: 7.1%; 95% CI: 6.5–7.7 to 6.8%; 95% CI: 6.3–7.3; *p* = 0.012) in a cross-over study of *n* = 27 adults with well controlled T2DM. Preliminary analysis of the AUSMED trial [33] for secondary prevention of CVD demonstrated significant reductions in subcutaneous adipose tissue (SAT) area compared to the low-fat diet control (MedDiet: −12.1 ± 6.5 cm^2^; low-fat: +0.4 ± 6.9 cm^2^; *p* = 0.04). However, no significant change in visceral adipose tissue (VAT) or any other body composition parameter was observed [34]. Interestingly however, despite significant improvements in adherence to a MedDiet pattern [35] no significant effect of the MedDiet was observed on makers of inflammation, oxidative stress, lipids, glucose or blood pressure when compared against a low-fat diet control [34].

In the MedLey study [36,37,38] adherence to a MedDiet intervention for 6 months resulted in significant reductions in systolic blood pressure (mean difference: −1.1 mm Hg; 95% CI: −2.0, −0.1 mm·Hg; *p* = 0.03), improved endothelial functioning (mean difference: 1.3%; 95% CI: 0.2, 2.4%; *p* = 0.03) and reductions in triglycerides (mean difference: −0.09 mmol/L; 95% CI: −0.18, −0.01 mmol/L; *p* = 0.03). However, no significant between group differences at any study time point were observed for lipoprotein profiles, glucose, insulin, c-reactive Protein (CRP) concentrations, BMI and waist-to-hip ratio. Similarly, in a randomized 2 × 2 crossover study design, results from the MedDairy study [39,40] revealed significant reductions in systolic blood pressure (mean difference: −3.51 mm·Hg; 95% CI: −6.35, −0.68 mmHg; *p* = 0.02), triglycerides (mean difference: −0.05 mmol/L; 95% CI: −0.08, −0.01 mmol/L; *p* < 0.01) and significantly higher HDL concentrations (mean difference: 0.04 mmol/L; 95% CI: 0.01, 0.06; *p* < 0.01) when compared against a low-fat control diet. In contrast, Wade et al. [41,42] reported no significant differences for blood pressure, lipids, glucose, insulin or CRP concentrations when a MedDiet intervention supplemented with 2–3 weekly serves of pork (MedPork study) was compared against a low-fat control diet.

In contrast, separate analyses from the MedDairy [39] and MedPork [41] studies revealed that a MedDiet intervention supplemented with either dairy or pork improved cognitive and psychological well-being when compared against a low-fat control diet [44,45]. Specifically, in the MedDairy study the investigators showed that adherence to a MedDiet supplemented with additional dairy foods significantly increased processing speed (*p* = 0.04) as assessed against the Cambridge Neuropsychological Test Automated Battery (CANTAB) and significantly reduced scores for total mood disturbance (*p* = 0.01), tension (*p* = 0.03), depression (*p* = 0.03), anger (*p* = 0.02) and confusion (*p* = 0.01) as assessed by the Profile of Mood States (POMS) questionnaire [44]. Similarly, in the MedPork study, adherence to a MedDiet intervention significantly increased processing speed (*p* = 0.01) as assessed against CANTAB and significantly increased scores for emotional role functioning when assessed against the SF-36 Health Survey for psychological well-being [45]. In contrast however, separate analyses from the MedLey study [36], revealed no evidence of a beneficial effect when adopting a MedDiet intervention on cognitive performance in healthy older adults [43].

A recent 12-week clinical trial [46] investigating the efficacy of an ad libitum MedDiet intervention versus a standard low-fat control diet on hepatic steatosis showed no significant changes between the two groups for reductions in hepatic fat in patients with NAFLD. However, significant reductions from baseline (all *p* < 0.05) were observed for total cholesterol, triglycerides and HbA1c in patients randomized to receive the MedDiet intervention [46].

Lastly, the SMILES [47,49] and HELFIMED [48,50] studies were one of the first clinical trials worldwide to demonstrate the efficacy of a MedDiet on mental health and depressive symtomology in adults with diagnosed depression. Specifically, in a 12-week single blinded RCT investigating the efficacy of a MedDiet intervention versus a social support control in participants with moderate to severe depression, Jacka et al. [47] reported significant reductions in depressive symptomology (*t*(60.7) = 4.38, *p* < 0.001; Cohen’s *d* = −1.16 (95% CI: −1.73, −0.59) in participants randomized to receive the MedDiet intervention. Similarly, in a 3-month single blinded RCT in participants with self-reported depression, Parletta et al. [48] reported that a MedDiet intervention supplemented with *n*-3 fish oil significantly reduced depressive episodes (*t* = −2.24, *p* = 0.03) and improved mental health QoL scores (*t* = 2.10, *p* = 0.04) at 3 months when compared against a social group control.

## 4. Assessing Adherence to a Mediterranean Diet in Clinical Trials Conducted in Australia: What is the Evidence?

Adherence to a MedDiet assessed in observational studies conducted in Australia in middle aged and older populations have reported low-moderate adherence at best [51,52,53,54,55]. However, all RCTs in the present review demonstrated impressive and sustained adherence to a MedDiet intervention. Specifically, analysis of the AUSMED trial [34,35] demonstrated that patients with pre-existing CVD achieved high adherence to the MedDiet following a 6-month intervention (4.8 ± 2.7-point increase in MEDAS score from baseline; *p* < 0.001; 95% CI: 3.9–5.6). Similarly, after 4 months of the intervention, a cohort of community-dwelling older adults recruited into the MedLey study demonstrated a 3.4-point increase (*p* < 0.001) in adherence to a MedDiet [37,38]. At completion of the intervention, results from the MedDairy study showed a 2.6-point increase in the MDS score amongst middle aged adults, translating to a significant increase in the consumption of food groups consistent with a MedDiet pattern including vegetables (*p* = 0.01), legumes (*p* < 0.001), nuts and seeds (*p* < 0.001), seafood (*p* = 0.02), dairy (*p* < 0.001) and EVOO (*p* < 0.001) [40]. Similar adherence trends were also reported amongst participants with depressive symtomology. In the HELFIMED study, after the completion of a 3-month MedDiet intervention, participants with self-reported depression significantly increased adherence scores (MEDAS baseline: 4.57 ± 0.24; 3-months: 7.08 ± 0.28; *p* < 0.001) [48].

Both the MedPork and SMILES studies assessed dietary compliance using a modified adherence tool that was adapted from the PREDIMED study [22] to best reflect the intervention protocols and studied population for each respective study. Specifically, in the MedPork study [42], consumption of ≥1 tbsp of EVOO was awarded 1 point, as compared to the necessary ≥4 tbsp required in the PREDIMED design [22]. Moreover, specific items related to fresh pork consumption were also added to reflect the dietary protocol [42]. In the SMILES study design [47], the modified MedDiet adherence tool was based on the consumption of key food groups including wholegrains, vegetables, fruits, legumes, nuts, fish, lean red meats, chicken, low fat dairy, eggs, olive oil and discretionary foods. Specific details pertaining to the scoring system were not reported by the investigators. Investigators from the MedPork study reported excellent compliance toward the dietary intervention (90% compliance) with the MedPork adherence score increasing 7.5 points from baseline [42]. At completion of the intervention, this translated to a significantly higher intake of % energy coming from total fat (*p* < 0.001), MUFA (*p* < 0.001), PUFA (*p* < 0.001) and MUFA:SFA (*p* < 0.001) in participants randomized to receive the MedPork intervention [42]. Upon completion of the SMILES study, participants randomized to receive the MedDiet intervention improved overall diet quality with a significant increase in the consumption of wholegrains (1.21 ± 1.77 serves/day), fruit (0.46 ± 0.71 serves/day), dairy (0.52 ± 0.72 serves/day), olive oil (0.42 ± 0.49 serves/day), legumes (1.40 ± 2.39 serves/week), and fish (1.12 ± 2.65) serves/week) [47]. Moreover, these changes in dietary behaviours also resulted in substantial reductions in the consumption of unfavorable discretionary food items (mean decrease: 21.8 ± 16.01 serves/week) [47]. Unlike previous studies that used specific MedDiet adherence index tools or modified versions of these, Itsiopoulos et al. [32] reported significant changes in plasma levels of several carotenoids including lycopene, lutein and zeaxanthin as biological markers for increased adherence to the MedDiet intervention.

Despite overall increases in adherence to a MedDiet throughout the intervention periods, the feasibility of ad libitum adherence in free-living populations is unknown. Moreover, it should be acknowledged that many of these studies used intensive, yet successful strategies to facilitate dietary compliance to the MedDiet intervention including regular contact via one-on-one personalized dietary prescription and counselling provided by an Accredited Practicing Dietitian, written resources (recipes, daily/weekly meal plans, shopping lists, label reading information), cooking classes, information sessions, pre-prepared meals and the provision of key ‘staple’ foods included in a traditional MedDiet such as EVOO, nuts and legumes. Nevertheless, it is arguable that such intensive strategies are necessary to facilitate dietary and behavior change in order to promote dietary compliance and principles of the MedDiet in non-Mediterranean countries. Perhaps most pertinently, the follow-up periods for the AUSMED [33], MedLey [36] and HELFIMED [50] studies all reported sustained high adherence to a MedDiet, even after the intervention and supportive adherence strategies had ceased. Specifically, in the AUSMED study [33], at the 12-month follow-up, adherence levels were sustained with 78% of participants achieving ‘high’ adherence scores compared with just 11.1% at baseline [35]. Similarly, at the 18-month follow-up of the MedLey study [36], adherence to a MedDiet amongst older adults remained unchanged with the consumption of EVOO, legumes, fish and vegetables all remaining higher relative to baseline levels [56]. Similar observations were also reported in the HELFIMED study [50] where healthy dietary behaviors were sustained 6-months post the MedDiet intervention [48].

## 5. Discussion

We reviewed the efficacy and adherence to a MedDiet adopted as a dietary intervention in clinical trials conducted in Australia on middle-aged to older adults against primary outcomes related to cardiometabolic risk factors, glycaemic control, cognition, hepatic steatosis and depressive symptomology. Across all RCTs included in the present review, we report impressive and sustained adherence to a MedDiet intervention. However, we report considerable differences in the effectiveness of the MedDiet as an intervention, particularly against cardiometabolic outcomes. We believe that this is likely due to the heterogeneity in the dietary protocols and prescriptive interpretation of a MedDiet across all studies presented in this review, making interpretations of the efficacy and adherence to the dietary pattern challenging.

All studies included in the present review identified their dietary intervention as a MedDiet; nonetheless, there was heterogeneity with respect to the definition and interpretation of a MedDiet as a prescriptive intervention. Specifically, the MedLey [36,37,38,43] and SMILES [47,49] studies modelled prescriptive MedDiet interventions that were comparable to the PREDIMED study [22] but adapted to be more aligned with the ADGs [57] and to increase palatability and acceptability for an Australian population. Similarly, the MedDairy [39,40,44] and MedPork [41,42,45] studies also adapted prescriptive MedDiet interventions derived from the PREDIMED study [22]; however, specific food items were added to reflect the dietary protocol and palatability and sustainability of the interventions. For example, in the MedDairy study [39,40,44], participants were advised to consume a MedDiet intervention which included 3–4 daily servings of dairy foods. Similarly, in the MedPork study [41,42,45], participants were advised to consume 2–3 weekly servings of fresh lean pork, and in preference to chicken and red meat. In contrast, the AUSMED [33,34,35] intervention was modelled on a 2-week meal plan which incorporated key dietary principles of a MedDiet and a mix of traditional and modified recipes to best reflect the multiethnic landscape of Australia [12]. The dietary interventions prescribed by Itsiopoulos et al. [32] and Properzi et al. [46] were based on a reconstruction of the traditional Cretan MedDiet [58]. Lastly, the HELFIMED [48,50] study did not include a dietary intervention per se; rather, participants were scheduled to attend fortnightly cooking workshops in a commercial kitchen, where recipes focused on simple, healthy, affordable and palatable meals using Mediterranean-style dietary principles. In contrast to all other studies included in the present review, participants randomized to the MedDiet group also received a 3-month supply of fish oil capsules (containing 450 mg DHA and 100 mg EPA) and were advised to consume 2 per day.

It is plausible that any differences observed in the efficacy of a MedDiet were attributable to discordance between dietary protocols, which may result in the displacement of bioactive nutrients and thus dilute any potential mechanistic benefit of the dietary intervention. This was potentially observed in the MedPork [42] study which, in contrast to the MedLey [37,38] and MedDairy [40] studies, reported no significant differences for cardiometabolic outcomes when compared against a low-fat control diet. Specifically, it is possible that the addition of pork to the MedDiet protocol diluted any potential health benefits of the MedDiet intervention given that pork is not a traditional dietary constituent of a traditional MedDiet [17,58]. Nevertheless, dairy is also not a traditional component of the MedDiet (unless fermented), and also receives a negative score in the 9-point MedDiet adherence score developed by Trichopoulou et al. [20]. Nevertheless, and perhaps most pertinently, the short study duration (8 weeks) possibly limited any chance of observing any significant improvements in cardiometabolic parameters related to health and disease prevention including cholesterol, glucose, insulin and inflammatory markers. Lastly, our review included studies of heterogeneous populations, ranging from otherwise healthy participants to those with pre-existing CVD-related risk factors, T2DM, depression and NAFLD. Therefore, it is not surprising that many of the cardiometabolic improvements were observed in already at-risk populations, which is a consistent finding within the literature [22,59,60,61]. In contrast however, Mayr et al. [34] reported that adherence to a MedDiet had no effect on LDL cholesterol, triglycerides or glucose when compared against a low-fat control diet in patients with pre-existing CHD. However, this finding was not unexpected given that most participants were prescribed statins or other lipid lowering medications as well as anti-hypertensives, and nearly all participants with T2DM were taking oral hypoglycaemic agents prior to study enrolment [34]. Of interest however, there was a significant reduction in the proportion of participants prescribed β-blockers (15%) amongst those participants randomized to receive the MedDiet intervention between baseline and 3-months, which was maintained at 6-months (*p*-trend = 0.007) [34].

Importantly a MedDiet is indeed prescriptive, both qualitatively (food components) and quantitatively (breakdown of macro and micronutrients). We recently reported considerable variation in the qualitative interpretation and quantitative prescription of a MedDiet when prescribed as a dietary intervention in clinical trials involving participants with T2DM [18]. Moreover, two previous systematic reviews have also described marked heterogeneity with respect to the definition and interpretation of a MedDiet [62,63]. Nevertheless, such differences are likely attributable to a continuous evolution of the diet, evolving from generation to generation in response to social, cultural, economic, environmental and historical circumstances [17]. Even in landmark clinical trials, there is discordance in the interpretation of a MedDiet. For example, the pioneering Lyon Diet Heart Study [64] and most recently, the PREDIMED study [22] were not traditional MedDiet interventions. In the Lyon Diet Heart Study, participants randomized to the MedDiet intervention were asked to replace butter and cream with an α-linolenic acid enriched margarine [64]. Exclusive use of olive oil was not recommended because it was not culturally accepted as the only oil source in the diet. Moreover, the PREDIMED study illustrated an example of a modified MedDiet given that the study design involved examining the efficacy of two MedDiets; one supplemented with EVOO and the other with mixed nuts [22]. Importantly, results from both studies were impressive with both trials stopping early with preliminary analysis indicating that the MedDiet intervention across both trials significantly reduced the incidence of primary [22] and secondary [64] cardiovascular events, respectively. However, the large heterogeneity around the definitions and interpretations of what constitutes a MedDiet necessitates for greater consistency and accuracy in the reporting and operationalization of a MedDiet for future investigations. This facilitates a shared understanding that allows for transparency when reviewing literature, identifying mechanistic benefits associated with the diet, as well as translation into practice-based guidelines, including the development and dissemination of dietary guidelines [12,18]. Despite the heterogeneity in the dietary protocols, in contrast to our previous findings [18], the reporting of MedDiet interventions across all studies included in the present review adequately described fundamental constituents of a MedDiet including the provision of fruits, vegetables, legumes, nuts, wholegrains, EVOO, fish, moderate alcohol consumption, preferential consumption of white meat, and a low consumption of red and processed meats, butter and discretionary foods. Although the exact mechanisms by which a MedDiet exerts its beneficial effects on preventing and managing NCDs is largely unknown, a number of interrelated and overlapping factors have been postulated including: (a) Lipid-lowering effect; (b) protection against oxidative stress, inflammation and platelet aggregation; (c) modulation of hormones and growth factors involved in the pathogenesis of disease; (d) gut microbiota-mediated production of metabolites influencing metabolic health [65]. Nevertheless, it is unknown whether these benefits are dose dependent, highlighting the need for greater consistency between studies in the types and amounts of foods that are recommended as a component of a MedDiet intervention.

Nevertheless, synthesis of the reviewed literature suggests excellent and sustained adherence to a MedDiet intervention across all studies. Although promising, it should be noted that many of the scoring systems used to evaluate dietary adherence are not homogeneous and differ between studies, making comparisons difficult. Unlike the MEDAS tool [21] which is based on normative criterion cut-off scores and reflective of a traditional MedDiet, the MDS is built by assigning a value of 0 or 1 to a total of nine dietary components with the use of gender-specific medians within the studied population as the cutoff [20]. Therefore, a potential limitation of the MDS, and other population-based adherence tools, is that it relies on sample medians and dependent on the habitual dietary characteristics of the studied population, potentially limiting its generalizability, particularly in non-Mediterranean populations [66].

The rationale for assessing the efficacy and adherence to a MedDiet in an Australian population is to identify a dietary pattern with a robust scientific evidence base that can prevent and manage the growing prevalence of NCDs. When considering the feasibility of adopting a MedDiet in an Australian population, it is generally easier to replicate the quantitative prescription of the dietary pattern (e.g., desirable macronutrient contribution) given that macronutrients are broad and non-specific [12]. The challenge however becomes multifaceted when we consider foods and their combination through cuisine, to replicate the synergistic effects of the food matrix [12]. Moreover, contextual dietary and lifestyle characteristics of a population should also be considered in the transferability of a MedDiet pattern to countries outside of the Mediterranean basin. For example, George et al. [12] recently demonstrated how key dietary principles related to traditional MedDiet cuisine could be replicated across a variety of traditional culturally-specific dishes. Specifically, the authors developed a MedDiet model that conformed with principles of a traditional MedDiet applied in a multiethnic context [12]. Akin to a traditional MedDiet [17,58], the MedDiet model was predominately a plant-based dietary pattern high in fat, mainly derived from EVOO and other MUFA sources. Moreover, it included moderate amounts of fermented dairy, fish and a preferential consumption of white meat over red meat [12]. Given that adherence to traditional MedDiet principles inevitably requires a low and infrequent consumption of red and processed meats [66], to ensure acceptability and palatability, the MedDiet model adopted a maximum quantity of 450 g/week (white and red meat varieties) which is consistent with ADG recommendations [57]. Although the MedDiet model resulted in slightly higher protein recommendations relative to a traditional MedDiet [17,58], adapting or modernizing a traditional MedDiet to satisfy the population’s nutritional requirements warrants further exploration. A recent example of this is demonstrated in the MedDairy study [39] whereby the MedDiet intervention was supplemented with additional dairy foods (3–4 serves per day) to adhere to ADG recommendations for dairy [57] and the recommended dietary intake for calcium [67], which would otherwise not be achieved with adherence to a traditional MedDiet [17,58].

Given that socio-cultural norms and palatability are important considerations for maximizing adherence to a dietary intervention [68], it is likely that non-Mediterranean populations such as Australia may be more likely to adhere to a diet containing more animal-based protein. Food and nutrient analysis from the most recent Australian Health Survey revealed Australian adults exceed ADG recommendations for red meat consumption by at least 25%, consuming on average 565 g per week, equivalent to 81 g per day [69]. Nevertheless, at the population level there is secular evidence suggesting a clear shift in the choice of poultry over red meat consumption [70]. Importantly however, processed meats (considered discretionary food choices because they are high in saturated fat and sodium) contribute ~10% of daily sodium intake in the Australian diet and make up ~20% of the population’s meat consumption, with the most commonly consumed processed meats being sausages, ham, bacon and luncheon meats [71]. Despite inconsistencies in the evidence related to the deleterious effects of red meat consumption on markers of cardiovascular health [72], a recent meta-analysis of RCTs reported that substituting red meat with high-quality plant protein sources results in favorable changes to blood lipids and lipoprotein profiles [73]. Moreover, recent findings from systematic reviews, meta-analyses and working group position statements suggest that a high consumption of red and processed meat is positively associated with the risk of T2DM [74,75], stroke [76,77], metabolic syndrome [78] and colorectal cancer [79,80]. Therefore, substituting red meat with other animal-derived protein rich foods may improve the palatability and acceptability of a Mediterranean-style diet in non-Mediterranean populations. This was demonstrated in the MedPork study [41] where participants were advised to follow a MedDiet intervention supplemented with 2–3 weekly serves of fresh, lean pork. Moreover, in a secondary analysis of the PREDIMED study [22], the investigators reported that replacing red meat and/or processed red meat with other animal-derived protein-rich foods, such as poultry, was associated with a lower risk of the metabolic syndrome [81].

The 2015–2020 Dietary Guidelines for Americans provides a successful platform of a population-wide adoption of key principles of the MedDiet, which includes an alternative healthy Mediterranean-style eating pattern, complemented with recommended amounts for each food group adapting for the American customary system [66]. When considering the previously established health benefits, in addition with the recently established environmental sustainability targets of food systems, it would be prudent for the next iteration of the ADGs to emphasize key dietary principles and/or food components of a MedDiet pattern, whilst preserving traditional culturally practices within the multiethnic landscape of Australia. By applying evidence-based knowledge and new policy strategies based on principles of the MedDiet, the next iteration of the ADGs has potential to facilitate lifestyle changes that are needed to improve the overall health status of Australia, both at the population level and environmentally. Nevertheless, a population-wide adoption of MedDiet principles requires a deliberate multisectoral approach with collaboration and commitment from the food industry, regulatory bodies, policy makers and health professionals alike [14].

## 6. Conclusions

The traditional MedDiet emphasizes a high-consumption of plant-based foods (fruits, vegetables, legumes and nuts) and EVOO as the principle culinary fat source, with an infrequent and low consumption of red/processed meats, butter and discretionary foods. The dietary pattern is consistent with the plant-based ‘planetary diet’ conveyed by the EAT-Lancet Commission report. However, whether non-Mediterranean countries such as Australia can adhere to a MedDiet is less clear. Synthesis of the reviewed literature from Australian RCTs involving middle-aged to older adults suggests excellent adherence, albeit with the use of intensive, yet successful strategies to facilitate dietary compliance. However, we report heterogeneity in the dietary protocols and prescriptive interpretation of a MedDiet across all studies presented in this review, making interpretations of the efficacy and adherence challenging. Nevertheless, with ongoing individualized medical nutrition therapy, counseling and behavioral modification strategies, evidence presented in this review should provide health care clinicians with confidence that adherence to key dietary principles of a MedDiet is feasible. Based on the observable health benefits, translating key dietary elements of a Mediterranean-style diet within the Australian population remains attractive. Despite the obvious barrier of its feasibility, acceptability and adaptability to the multiethnic landscape of Australia, this paper provides evidence and insight into the possibility of translating key dietary principles of the MedDiet which could be used by policy makers and regulatory bodies in the next iteration of the ADGs. Lastly, with an increased focus on consuming sustainable diets, the MedDiet provides a viable and appetizing tested diet.

## Figures and Tables

**Table 1:** Characteristics of included studies related to the efficacy of a Mediterranean Diet in randomized control trials conducted in Australia.

Author	Primary Aims	Study Population	Study Duration	Sample Size	Control Diets	Primary Outcomes
Itsiopoulos et al. [32]	To examine the efficacy of a traditional Mediterranean-type cuisine on HbA1c and vascular risk.	Participants with well-controlled T2DM	24-weeks (cross-over study; no wash out period used)	27	Habitual diet	Adherence to a traditional moderate-fat Mediterranean diet improves glycemic control and diet quality in patients with well-controlled T2DM.
Mayr et al. [34,35]	To determine the efficacy of an ad libitum MedDiet on cardiometabolic risk markers	Participants with pre-existing CHD	6-months	65 MedDiet *n* = 34; Low-fat *n* = 31	Low-fat diet; standard low-fat diet recommendations consistent with ADG and National Heart Foundation recommendations	Adherence to the MedDiet intervention significantly reduced SAT but not VAT area. No significant between group differences were observed on markers of inflammation, oxidative stress, lipids, glucose and BP.
Davis et al. ^1^ [37,38] Knight et al. ^2^ [43]	1. To assess the impact of a MedDiet pattern on BP and endothelial function in an older Australian population 2. To examine the examining effect of a MedDiet pattern on cognitive function	Otherwise healthy older adults aged ≥64 years	6-months	166 MedDiet *n* = 85; Habitual diet *n* = 81	Habitual diet	1. Adherence to a MedDiet intervention resulted in a small but significantly lower systolic blood pressure and improved endothelial function. However, no significant between group differences observed for lipoprotein profiles, glucose, insulin, CRP, BMI and waist-to-hip ratio. 2. No evidence of a beneficial effect for a MedDiet intervention on cognitive performance.
Wade et al. ^1^ [40] Wade et al. ^2^ [44]	1. To determine the effect of a MedDiet intervention supplemented with dairy foods on cardiovascular risk factors 2. To determine the cognitive and psychological effects of a MedDiet supplemented with dairy foods	Males and females aged between 45–75 years with at least 2 CVD risk factors	24-weeks (cross-over study; participants followed each intervention for 8-weeks with an 8-week washout period separating the interventions)	41	Low-fat diet; standard low-fat diet designed to replicate the control diet used in the PREDIMED trial [8]	1. Adherence to a MedDiet intervention supplemented with additional dairy foods led to significant changes in markers of cardiovascular risk in participants at risk of CVD. 2. Adherence to a MedDiet supplemented with additional dairy foods led to improvements in mood and processing speed in participants at risk of CVD.
Wade et al. ^1^ [42] Wade et al. ^2^ [45]	1. To assess the cardiovascular effects of a MedDiet intervention supplemented with fresh, lean pork 2. To assess a MedDiet supplemented with 2–3 weekly servings of fresh lean pork against measures of cognitive function and well-being	Males and females aged between 45–80 years with at least 2 CVD risk factors	24-weeks (cross-over study; participants followed each intervention for 8-weeks with an 8-week washout period separating the interventions)	33	Low-fat diet; standard low-fat diet designed to replicate the control diet used in the PREDIMED trial [8]	The MedPork intervention resulted in no significant between group differences for blood pressure, lipids, glucose, insulin or CRP concentrations. Compared with the low-fat control diet, the MedPork intervention led to higher performance in the cognitive domain of processing speed and higher scores for the SF-36 subscale, emotional role functioning.
Properzi et al. [46]	To examine the efficacy of an ad libitum MedDiet on hepatic steatosis and cardiometabolic risk factors	Adult patients with a diagnosis of NAFLD	12-weeks	48 MedDiet *n* = 48; Low-fat *n* = 48	Low-fat diet; standard low-fat diet recommendations consistent with ADG and American Heart Foundation recommendations	An ad libitum MedDiet intervention showed no significant between group differences in hepatic steatosis and measures of liver function. However, the MedDiet intervention lead to significant reductions in total cholesterol, triglycerides and HbA1c
Jacka et al. [47]	To investigate the efficacy of a MedDiet intervention for the treatment of major depressive episodes	Males and females with moderate to severe depression	12-weeks	67 MedDiet *n* = 33; Social support *n* = 34	Social support group; nil dietary intervention	Adherence to a MedDiet intervention resulted in significant reductions in depressive symptomology, independent of any changes in BMI, self-efficacy, smoking rates and/or physical activity
Parletta et al. [48]	To investigate the efficacy of a MedDiet supplemented with *n*-3 fish oil on mental health and depressive symtomology	Males and females with self-reported depression	6-months (3-month intervention, with 3-month follow-up)	152 MedDiet *n* = 75; Social support *n* = 77	Social support group; nil dietary intervention	Adherence to a MedDiet intervention supplemented with *n*-3 fish oil significantly reduced depressive episodes and improved mental health QoL scores

## Abbreviations

MedDiet	Mediterranean Diet
T2DM	Type 2 diabetes mellitus
CHD	coronary heart disease
SAT	subcutaneous adipose tissue
VAT	visceral adipose tissue
BP	blood pressure
CRP	C-reactive protein
CVD	cardiovascular disease
ADG	Australian Dietary Guidelines
SF-36	self-administered short-form 36 health survey
NAFLD	non-alcoholic fatty liver disease
HbA1c	glycated haemoglobin
BMI	body mass index
*n*-3	omega-3
QoL	quality of life
